# Reviewed article: Mota DM, Leitão LO, Silva RS, Fernandes CFR, Silva MT. Actions to mitigate ocular adverse events related to hair styling cosmetics in Brazil: a descriptive and correlational study (2022-2024). Epidemiol Serv Saude. 2025:34;e20240449

**DOI:** 10.1590/S2237-96222025v34e20240449.c

**Published:** 2025-05-02

**Authors:** Maryana Carmello da Costa

**Affiliations:** FOUSP

**Table:** 

Rating	Excellent	Good	Average	Below average	Poor
Interest		✓			
Quality		✓			
Originality		✓			
Overall		✓			

**Table:** 

Questionnaire	Yes	No	Not applicable
Does the manuscript contain new and significant information to justify publication?	✓		
Does the Abstract (Summary) clearly and accurately describe the content of the article?		✓	
Is the problem significant and concisely stated?	✓		
Are the methods described comprehensively?	✓		
Are the interpretations and conclusions justified by the results?	✓		
Is adequate reference made to other work in the field?	✓		

## Manuscript Structure:

Length of article is: Adequate

Number of tables is: Adequate

Number of figures is: Adequate

Please state any conflict(s) of interest that you have in relation to the review of this paper (state “none” if this is not applicable): None

## Comments

Em relação a tabelas e figuras, é necessário checar o Quadro 1. Fontes de dados, tipos de documentos e estratégias de buscas dos documentos utilizados nesse estudo em relação a erros ortográficos.

Em relação ao resumo, revise os métodos e conclusão. Descreva com mais clareza os métodos e apresente uma conclusão clara que respond ao objetivo.

O trabalho apresenta-se relevante, considere a importância de destacar a relevância do trabalho para o campo de pesquisa no início da discussão

Descreva quais foram os vieses, os procedimentos empregados no estudo para minimizá-los. 

